# Comparison of the Rapid Antigen Testing Method With RT-qPCR for the Diagnosis of COVID-19

**DOI:** 10.7759/cureus.17405

**Published:** 2021-08-24

**Authors:** Ashok Kumar Pandey, Aroop Mohanty, Vivek Hada, Rama S Rath, Subodh Kumar, Surekha Kishore, Rajni Kant

**Affiliations:** 1 Virology, Indian Council of Medical Research-Regional Medical Research Centre, Gorakhpur, IND; 2 Clinical Microbiology, All India Institute of Medical Sciences, Gorakhpur, IND; 3 Community Medicine, All India Institute of Medical Sciences, Gorakhpur, IND; 4 Pulmonary Medicine, All India Institute of Medical Sciences, Gorakhpur, IND; 5 Medicine, All India Institute of Medical Sciences, Gorakhpur, IND; 6 Infectious Disease, Indian Council of Medical Research-Regional Medical Research Centre, Gorakhpur, IND

**Keywords:** rdt, covid-19, immunochromatography, test characteristics, rt-qpcr

## Abstract

Background: Coronavirus disease 2019 (COVID-19) has till now affected about 110 million people globally. It has not spared any country and has led to 24 lakh deaths. As a result, the testing had to be increased manifold leading to depletion in the number of the quantitative reverse transcription polymerase chain reaction (RT-qPCR) kits. Point-of-care rapid antigen-based tests were developed in order to meet the increasing demands. The objective of this study was to compare the performance of a rapid chromatographic test (index test) with a gold standard test (RT-qPCR).

Methods: A retrospective analysis was done at a tertiary care teaching hospital in Eastern Uttar Pradesh, India. Paired samples were taken from all patients reporting to the clinic for antigen-based rapid diagnostic testing (RDT) and RT-qPCR. The sensitivity and specificity were calculated to evaluate the performance of the RDT.

Results: The overall sensitivity and specificity of the RDT were observed to be 53.6% (39.7-67.0) and 97.35% (94.6-98.9), respectively. In symptomatic individuals, the sensitivity was higher 61.0% (44.5-75.8). The test positivity rates of RDT were found to be higher at a ​​cycle threshold value ≤20.

Conclusion: RDT can be used as a screening test to rule in the infection especially in symptomatic patients who are more prone to spread the disease. It is an important weapon in the armamentarium of public healthcare for the containment of COVID-19.

## Introduction

Coronaviruses are enveloped non-segmented, positive-sense RNA viruses belonging to the family *Coronaviridae* and order Nidovirales [1,2]. They cause multiple system infections in animals but mainly lead to respiratory tract infections in human beings [3,4]. Although most of the human infections are mild in nature, the epidemics of the other two betacoronaviruses, severe acute respiratory syndrome coronavirus (SARS-CoV) and Middle East respiratory syndrome (MERS)-CoV, were fatal in nature and have caused more than 10,000 deaths in the past two decades. In December 2019, one more coronavirus disease emerged in Wuhan, China, and rapidly spread all over the world. After performing a deep sequencing analysis of the lower respiratory samples, this unknown virus was named as SARS-CoV-2 and disease named as coronavirus disease 2019 (COVID-19) [5,6]. Till date, India has reported 31,440,951 confirmed cases of COVID-19 and is ranked second only to the United States of America [7]. This growing trend and overwhelming increase in the total number of cases has overburdened our testing capacity and has led to a severe scarcity of molecular testing kits and reagents. Moreover, the quantitative reverse transcription polymerase chain reaction (RT-qPCR) testing requires a sophisticated biosafety level (BSL-2/BSL-3) laboratory and skilled technicians to perform the test. It takes a minimum of 8-10 hours for the generation of report from the receiving of the sample. Although it is the gold standard test for the diagnosis of COVID-19, it cannot be performed in each and every city/district due to lack of molecular virology facilities and difficulty in procuring reagents/viral transport medium (VTM) [8]. In view of the above, the need of the hour was to develop a point-of-care test that would detect and isolate positive cases rapidly, diagnose them at an early stage and contain the spread. This could prove very useful in an emergency department where a quick triage could be done for patients with severe acute respiratory illness (SARI). The healthcare workers (HCWs) are the most vulnerable group of people having the highest risk of contact with COVID-19. India alone has reported around 200 deaths of HCWs, which includes 64 doctors. In view of the resumption of outpatient department (OPD) services in our institute and the huge load of patients, we felt the need to start with the antigen-based rapid diagnostic testing (RDT) modality. The first of these to be validated by the Indian Council of Medical Research (ICMR) was the STANDARD Q COVID-19 antigen detection kit manufactured by SD Biosensor in South Korea [9]. However, only one study has been done in India to compare the performance and diagnostic utility of this test. Here, we report the evaluation of this rapid chromatographic test for the detection of SARS-CoV-2 antigen in comparison to RT-qPCR for the diagnosis of COVID-19 in India.

## Materials and methods

This is a retrospective analysis conducted in a tertiary care hospital in the eastern part of Uttar Pradesh, India. The main objective was to compare the recently approved rapid antigen test (RAT) with the gold standard RT-qPCR test.

During the unlocking phase in India, various activities were started gradually. To prevent the spread of the disease and for the early detection of COVID-19, a screening desk along with a flu clinic was established at the institute (​All India Institute of Medical Sciences, Gorakhpur, Uttar Pradesh). HCWs who were already working reported to the flu clinic if any symptoms related to COVID-19 were present.

RAT was conducted for all the patients visiting the flu clinic after the assessment of the symptoms using the STANDARD Q COVID-19 Ag (SD Biosensor) test kit. The test was performed as per the manufacturer's instruction, and is briefly discussed below. Additionally, irrespective of the result of RAT, nasopharyngeal and throat swabs was also collected for RT-qPCR using nylon flocked swabs and were placed in a 3-ml VTM tube (Viral Transport Kit III; Microexpress®, Verna, Goa, India) with proper identification. All the samples collected in the VTM were sent to the referral center, i.e., ICMR-Regional Medical Research Centre, Gorakhpur, for RT-qPCR maintaining the cold chain. All the samples were tested using ICMR-approved kits as per the protocol. The results of rapid antigen tests were not informed to the referral center and thus they were kept blinded. The patients who were found positive in either RAT or RT-qPCR were advised isolation and further management. Contact tracing of the staff was also conducted after the detection of a positive result. The high-risk contacts were tested after five days of the contact with laboratory-confirmed cases or development of symptoms, whichever was earlier.

For this study, the data of all such subjects who were tested using RAT was retrieved after proper Institutional Human Ethics Committee (IHEC) approval (IHEC/AAIMS-GKP/BMR/36/2020). The following group of subjects were included in our analysis: (1) all HCWs who were symptomatic for COVID-19 infection and visited the screening desk; (2) all asymptomatic/pre-symptomatic high-risk contacts of laboratory-confirmed cases identified during contact tracing.

RAT antigen extraction

All patients were asked to clear nasal secretions before the collection of samples. A sterile nasopharyngeal swab provided with the kit was inserted into the nasal cavity at an angle of 90° in an extended neck position to collect the swab from the posterior part of the nasopharynx. It was kept in the nasopharynx for about five seconds and was removed gently while rotating it. After this, the swab was inserted into the extraction buffer tube, provided with the kit and was dipped into it five to six times before squeezing it finally. The swab was discarded with full precaution. A nozzle was placed tightly to close the extraction buffer tube; it was shook for about 20 seconds and then two drops were put onto the specimen well of the test device given.

Interpretation of RAT results

The test result was interpreted after 15 minutes. The test device contains two lines: ‘C’, a control line, and ‘T’, a test line. If a red band formed at both ‘C’ and ‘T’ positions, the test was taken as positive. All red bands, whether they were faint or light at the ‘T’ position, were taken as positive. If the red band was formed only at the ‘C’ position, it was interpreted as negative whereas it was considered invalid if the band was not formed at the ‘C’ position. A repeat test was performed in such situations. As it is known that RT-qPCR has the highest sensitivity for the detection of SARS-CoV-2-specific gene targets, it was considered as the reference standard for the purpose of comparison [10].

## Results

A total of 321 suspected individuals were tested for rapid antigen testing and RT-qPCR simultaneously. From these 321 individuals, three samples showed indeterminate results in RT-qPCR and were thus excluded from the final analysis (n=318). The mean age of the suspected individuals was 29.4 years (SD ±10.11 years). Around 81% of the suspected were male and rest were female; 41% of the suspected presented with one or other symptoms whereas others were tested as a part of the contact tracing or as a part of screening before joining work or studies. From all tested samples (n=318), a total of 56 came out to be RT-PCR positive and 37 came out to be RAT positive. Thus, the prevalence of the COVID-19 in the study population was 17.6% (95% CI: 13.8%-22.2%) making RT-qPCR as the gold standard of diagnosis. The agreement between the tests was found to be moderate with a kappa value of 0.57 (p value: 0.000).

**Figure 1:**
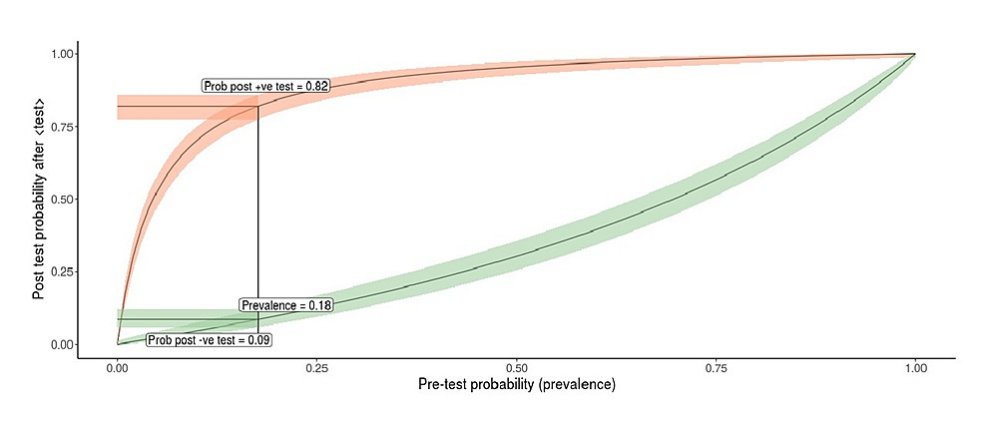
Relation between the pretest probability and posttest probability for the rapid antigen test in COVID-19 COVID-19, coronavirus disease 2019

The overall sensitivity and specificity of the antigen test came out to be 53.6% (95% CI: 39.7%-67.0%) and 97.3% (95% CI: 94.6%-98.9%), respectively. The positive predictive value (PPV) was 81.1% (95% CI: 64.8%-92.0%) and negative predictive value (NPV) 90.7% (95% CI: 86.7%-93.9%). The detailed test characteristics are represented in Table 1. The relation between the pretest probability and posttest probability is represented in the Figure 1. When tested individuals were segregated according to the symptoms, sensitivity of the RAT was found to be higher among the symptomatic individuals than the asymptomatic individuals whereas the specificity was found to be higher in asymptomatic individuals than the symptomatic. Thus, the RAT is a better test to rule out the infection than diagnosing the infection. This is also evident from the NPV value.

**Table 1:** ​Test parameters according to the type of patient (symptomatic vs asymptomatic) ​ LR, likelihood ratio; PPV, ​positive predictive value; NPV, ​negative predictive value

​	​Overall	​Symptomatic	​Asymptomatic
True positive	30	25	5
True negative	255	85	170
​False positive	7	5	2
​False negative	26	16	10
​Sensitivity	​53.6% (39.7-67.0)	​61.0% (44.5-75.8)	​33.3% (11.8-61.6)
​Specificity	​97.3% (94.6-98.9)	​94.4% (87.5-98.2)	​98.8% (95.9-99.9)
​LR (+)	20.1 (9.3-43.3)	11.0 (4.5-26.6)	28.7 (6.1-135.0)
​LR (-)	0.5 (0.4-0.6)	0.4 (0.3-0.6)	0.7 (0.5-1.0)
​PPV	81.1% (64.8-92.0)	83.3% (65.3-94.4)	71.4% (29.0-96.3)
​NPV	90.7% (86.7-93.9)	84.2% (75.6-90.7)	94.4% (90.0-97.3)

**Figure 2:**
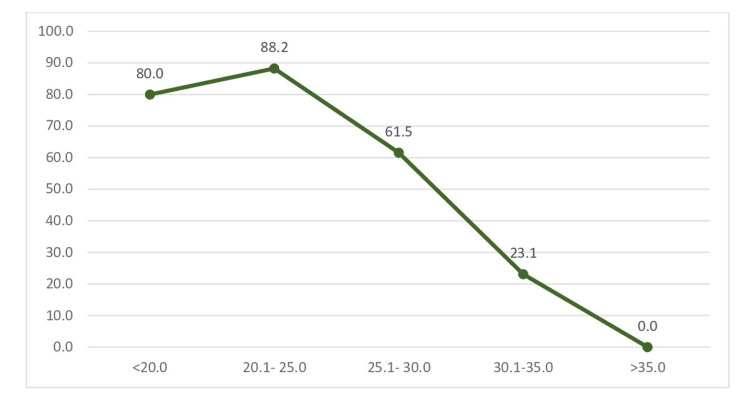
True positive rate of the ​rapid diagnostic test at different ​cycle threshold values

When we compared the test positivity at different ​​cycle threshold (CT) values, we found that the antigen positivity increases from CT values <20 to 21-25 to achieve a highest value of 88% and then decreases gradually to almost 0% in patients with a CT value more than 35 (Figure 2).

## Discussion

This study was a hospital-based study of the analysis of test characteristics of the rapid antigen test kit (STANDARD Q) when compared with the RT-qPCR (gold standard). The sensitivity of STANDARD Q was found to be 53.6% and specificity was found to be 97.3% with the PPV of 81.1% and NPV of 90.7%. Among symptomatic individuals, the sensitivity was found to be 61.0% and specificity was found to be 94.4% whereas among asymptomatic individuals, it was 33.3% and 98.8%, respectively.

Many studies conducted in India and abroad found the sensitivity and specificity to be higher as compared to the current study [11-15]. The reported sensitivity varied from as high as 93.9% to as low as 70.0% and the specificity varied from as high as 100% to as low as 92.0%. The severity of infection or antigen load may also be a critically determining factor. In the current study population, only 38.6% were symptomatic. When we classified the RT-qPCR results according to the CT values, we found the CT values ranged from 18 to 39. Only 5 cases (8.9%) reported to have CT values ≤20. Around 37.5% of the RT-qPCR-positive individuals had the CT values ≥30. When we compared the test positivity rate of RT-qPCR at various cutoffs of CT values, we found the test positivity to rapidly decline after the CT value of 25.1 to 30.0 from 88.2% to 61.5% (Figure 2). The test positivity further declined to 0% when the CT value was more than 35.0. This indicates the infection was low grade that might have resulted in the low sensitivity of the test results. A similar pattern was observed in the study conducted by Gupta et al. in New Delhi [15]. This indicates the antigen test is not a reliable in low-viral-load cases many of which are asymptomatic. Thus, rapid antigen test kits can be used as a screening test to rule out the infection but has lower significance when used in contacts who are asymptomatic. At the same time, in individuals with a high viral load, the sensitivity reaches as high as 88%. Thus, it can be used to screen the individuals with a high viral load (more likely to be symptomatic), thus preventing the spread of the disease in the community. The low sensitivity and specificity may be attributed to the low prevalence in the current study population as compared to the other studies. Although there is a wide variability in the sensitivity and specificity of the RDT, the kits are suitable for point-of-care testing and thus can have wide applicability in pandemic control [15].

The ​likelihood ratio was found to be around 20, which indicates there is a 20-fold increase in the likelihood of being COVID positive after RDT positivity. The posttest positivity increases from a pretest value of 18% to 82%, which shows that 82% times a person who is RDT positive is likely to be COVID positive, and 9% times, the RDT-negative patients may be COVID positive. Thus, highly susceptible cases like symptomatic contacts and cases from high-prevalence areas like hospital settings can be used to diagnose the cases that can be further confirmed by the RT-qPCR.

The study is strengthened by the fact that this is one of the few studies conducted in India that reported the test characteristics of the RDT kit for COVID-19. The study is limited by the fact that sample size has not been calculated for the study.

## Conclusions

The rapid antigen test is an important tool for controlling the ongoing COVID-19 pandemic and early detection of cases, particularly in special situations. It was seen in this study that the rapid antigen test performed well in the patients with a low CT value, i.e., a high viral load. Thus, all such cases if diagnosed early can help in breaking the chain of transmission. There is further need to assess the performance of various types of SARS-CoV-2 rapid antigen tests available commercially.
